# Synchronous Inflammatory Myofibroblastic Tumor in Lung and Brain: A Case Report and Review of Literature

**DOI:** 10.7759/cureus.1183

**Published:** 2017-04-20

**Authors:** Maham Jehangir, Aisha Jang, Imaad Ur Rehman, Nadira Mamoon

**Affiliations:** 1 Radiology, Shifa International Hospital, Islamabad, Pakistan; 2 Pathology, Shifa International Hospital, Islamabad, Pakistan

**Keywords:** inflammatory myofibroblastic tumor, lung, brain

## Abstract

Inflammatory myofibroblastic tumor (IMT) is a rare entity and a diagnostic challenge with myriad clinical presentations and pathogenetic mechanisms. Isolated occurrences can be at any site in the body; however, only a few cases of IMT with the concomitant appearance of different organs have been published. We report a unique occurrence of bilateral inflammatory myofibroblastic tumor of the lungs with synchronous brain parenchymal lesions in an 11-year-old male, detailing clinical presentation with the emphasis on imaging and review of the literature.

## Introduction

Inflammatory myofibroblastic tumor (IMT) is a rare tumor-like condition that may occur anywhere in the body but is most commonly found in the lungs. It accounts for less than one percent of all tumors of the lung and airways [1]. It has a predilection for the pediatric population. Alternate terminologies for IMT include pulmonary pseudotumor, plasma cell granuloma, fibroxanthoma, xanthomatous pseudotumor, and plasma cell-histiocytoma complex. Typical lesions are solitary and circumscribed. Clinical behavior varies widely, ranging from benign lesions with the favorable outcome to large masses of local invasion, the risk of distant metastasis and poor prognosis. It is rarely found in the head and neck except for the orbits. A cough, fever, dyspnea, and hemoptysis are the usual presenting symptoms [2]. Very few cases of pulmonary IMT with a concomitant appearance in the brain have been documented in the international literature [3-5]. We add to this short list of synchronous inflammatory myofibroblastic tumors involving brain and both lungs. An 11-year-old male presented with isolated neurological symptoms. Although large solid masses were found in both lungs, the patient had mild pulmonary symptoms at presentation.

We describe clinical, histopathological and imaging features of this patient and review the characteristics of co-existent intracranial and pulmonary IMTs reported in the literature. Informed consent statement was obtained for this study.

## Case presentation

An 11-year-old male presented to the emergency department (ED) with fever and seizures. He had seizures since the age of seven years and was first investigated at the age of nine for recurrent seizures. Initial imaging workup showed multifocal brain lesions and large left lung mass. Considering the positive history of tuberculosis contact and constitutional symptoms, the patient received anti-tubercular drugs for nine months without any symptomatic relief. He had a mild cough and exertional dyspnea at presentation; however, the seizure frequency had progressively worsened over two years. Examination showed a lean, afebrile child with pallor. Chest examination revealed vesicular breath sounds with reduced intensity of breath sounds and dull percussion on left side. Review of blood work showed microcytic hypochromic anemia, elevated RBC sed rate levels (ESR) of 140 mm/ hr and C-reactive protein (CRP) of 142 mg/ dL. 

**Figure 1:**
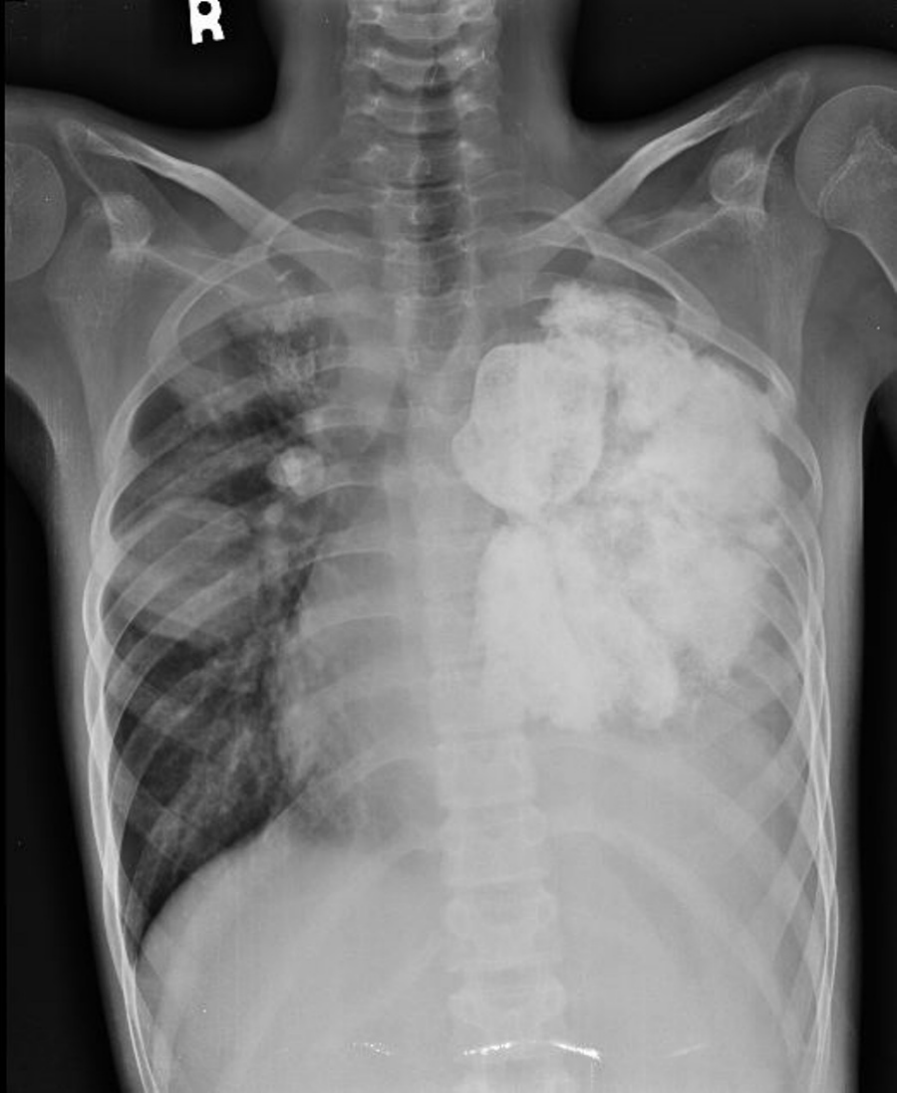
Chest radiograph Chest radiograph showing a large densely calcified left lung mass and additional small nodular opacities in the right lung mid zone, a few of which show central chunky calcifications

**Figure 2:**
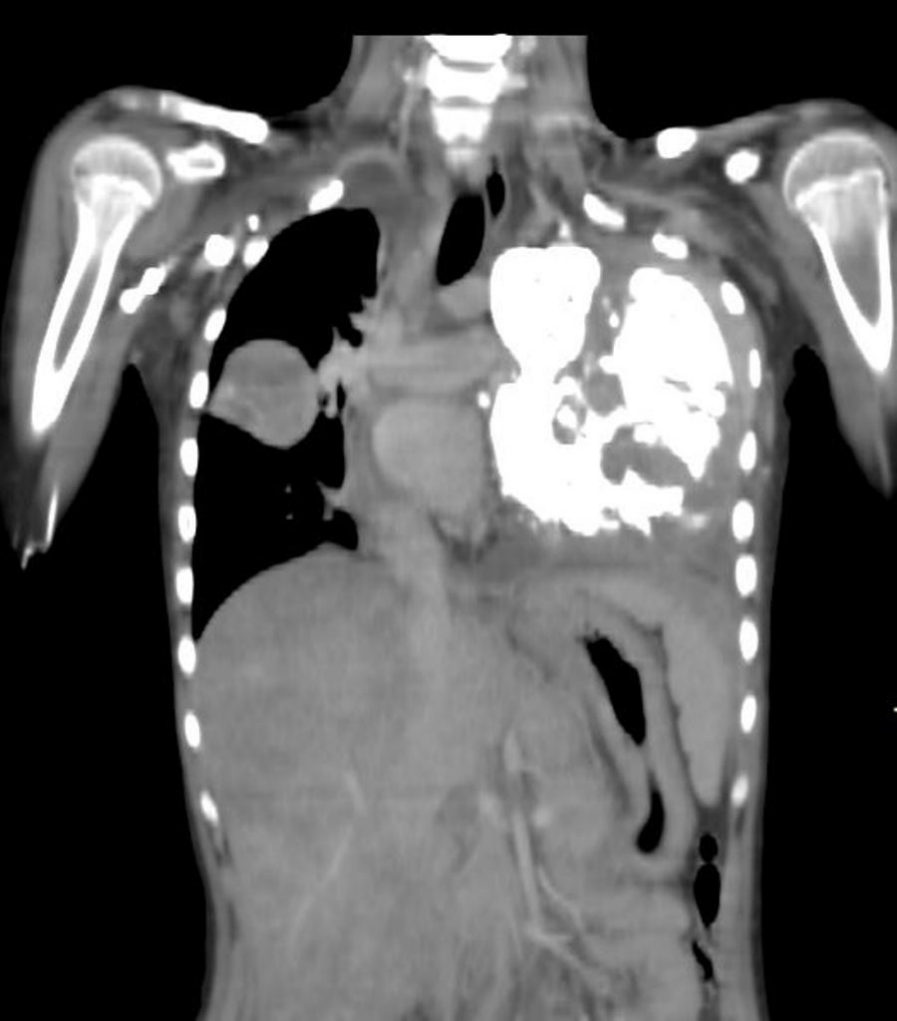
Contrast-enhanced coronal reformatted computed tomography (CT) scan A large calcified mass is seen in left hemithorax causing righward mediastinal displacement. It is inseperable from the mediastinum and the pleura. Left pulmonary vessels were narrowed and the left main bronchus was occluded (not shown). Multiple smaller partially calcified lesions are also seen in the right lung. The liver is enlarged

**Figure 3:**
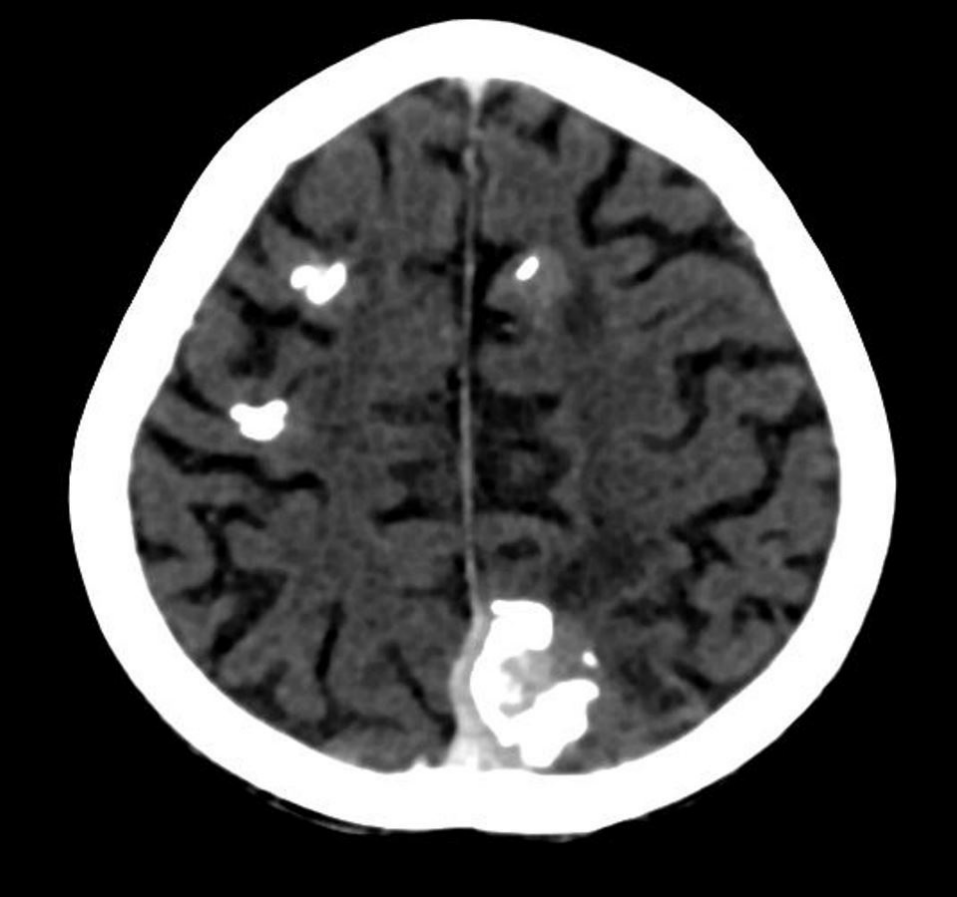
Contrast-enhanced axial CT brain Multiple solid enhancing cortical lesions with dense gyriform calcifications are seen in both cerebral hemispheres. CT- computed tomography

**Figure 4:**
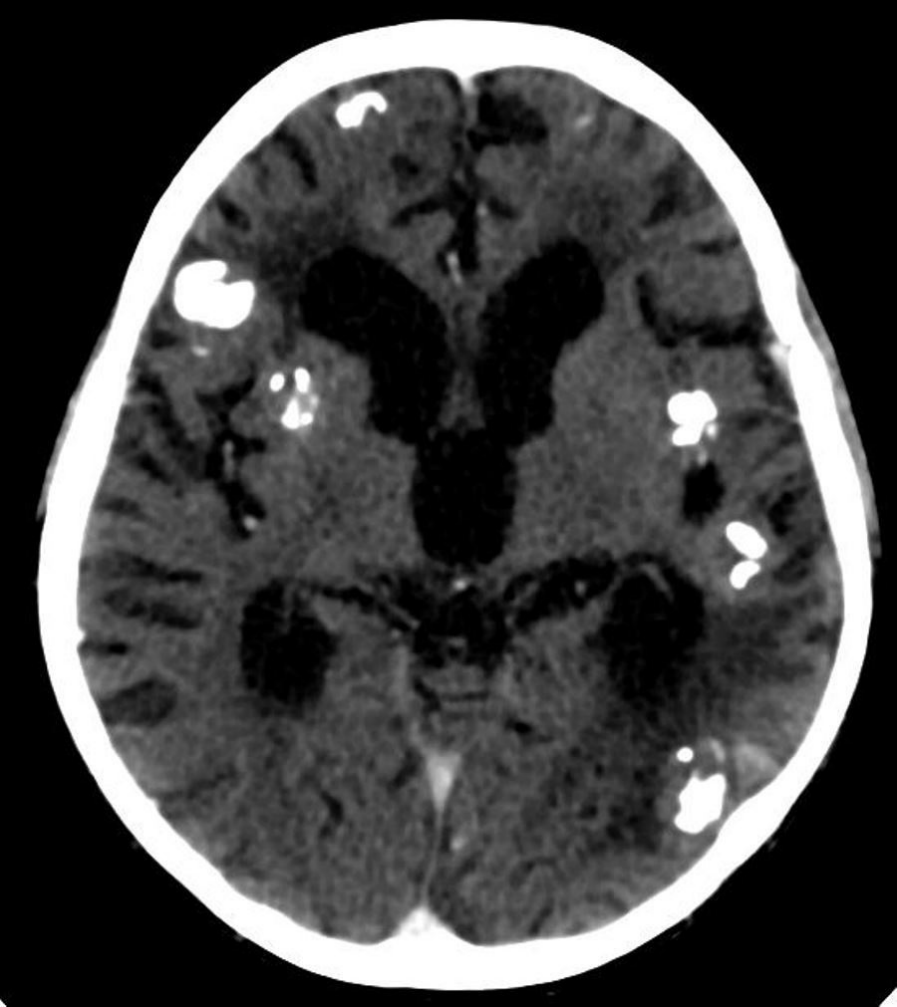
Contrast-enhanced axial CT brain Hydrocephalus and cortical atrophy are evident. Multiple calcified enhancing cortical lesions are also present. CT- computed tomography

Chest radiograph (Figure 1) and computed tomography (CT) scan of the chest showed a large [9 x 11 cm] lobulated soft tissue density solid mass in the left lung with large chunks of scattered popcorn like calcifications causing severe narrowing of left pulmonary vessels and occlusion of left mainstem bronchus. Multiple smaller partially calcified lesions were also seen in the right lung. Few of the lesions were also abutting the pleural surface. Bilateral pleural and pericardial effusion was also seen (Figure 2). Computed tomography scan of the brain showed multiple circumscribed calcified and non-calcified lesions with gyriform calcifications diffusely involving bilateral cerebral hemispheres (Figure 3). Largest lesion in left posterior parietal lobe measured 2 x 3 cm. Some of the lesions showed post contrast enhancement (Figure 4). Associated cortical atrophy and mild hydrocephalus were also present. There was no leptomeningeal enhancement.

**Figure 5:**
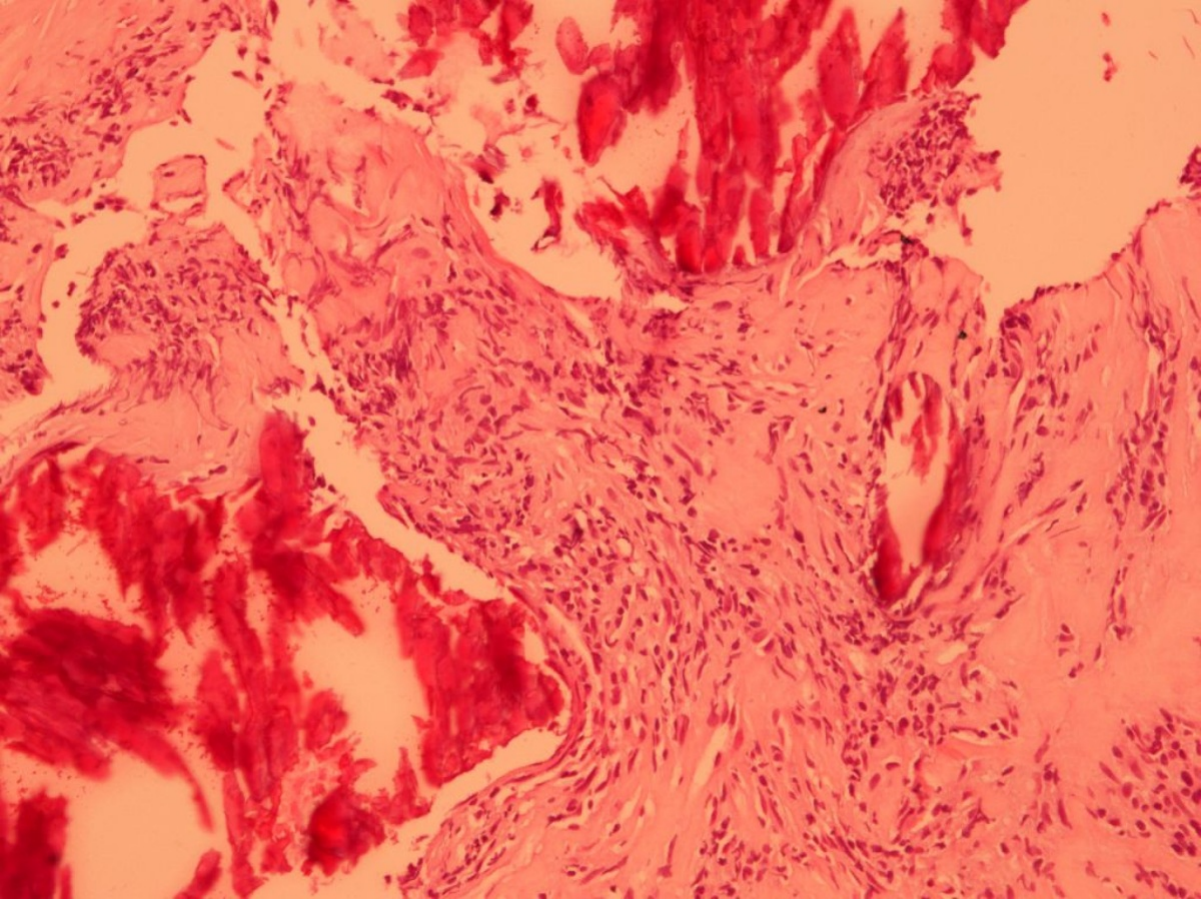
Photomicrograph of pulmonary mass biopsy Spindle cells arranged as fascicles. Areas of dense fibrosis and calcification are also present

The patient underwent ultrasound guided core needle biopsy of the left pulmonary lesion; the histological examination confirmed IMT. Fragments and cores comprising of a prominent component of inflammatory cells, particularly plasma cells and lymphocytes, admixed with bland spindle cells were seen. The spindle cells were arranged in fascicles with no hemorrhage, mitoses or necrosis. Areas of dense fibrosis and calcifications were also present (Figure 5). Immunohistochemistry showed kappa and lambda positivity in plasma cells. The anaplastic lymphoma kinase (ALK) was negative.

The case was discussed in a multidisciplinary meeting. As the lesion was encasing the major mediastinal vessels, it was considered inoperable. Symptomatic treatment was given on outpatient basis.

## Discussion

IMT is a quasi-neoplastic lesion consisting of inflammatory cells and myofibroblastic spindle cells. The origin, etiology, and behavior of IMTs remain a matter of debate. Originally considered a benign pathology, it is now clear that IMTs can have an aggressive behavior with an adverse prognosis [2]. Both benign and aggressive forms have similar morphologic features characterized by fascicles of bland myofibroblasts admixed with a prominent inflammatory component. The etiopathogenesis of IMT is poorly understood. Proposed mechanisms include trauma, surgical inflammation, immune-autoimmune condition, and infection. Their tendency to be locally aggressive, multifocal, and to progress occasionally to a true malignant tumor supports the neoplastic nature. IMTs have been linked to activin-like kinase anaplastic lymphoma kinase (ALK) gene (2p23) rearrangements [6], and some have suggested an association with the human herpes virus 8 (HHV-8) [6]. In recent years, studies of ALK 1 protein using immunohistochemical methods have shown positive staining in 50% cases [7]. IMT in the central nervous system (CNS) is rare. It can occur as an isolated entity or associated with similar tumors in another organ, usually mediastinum, lung or mesentery.

On reviewing literature, we found very few cases of combined pulmonary and intracranial inflammatory myofibroblastic tumors [5]. The most frequent pulmonary symptoms in these patients included a cough and hemoptysis. Intracranial symptoms included headache, vomiting, and seizures. Most of the cases were of pediatric age. Lung lesions in these cases were large solitary masses and brain lesions were small, mostly multifocal and discrete, suggesting the possibility of hematogenous dissemination [3-5]. In most cases, detection of lung lesions either preceded the brain lesion (mean duration from five months to five years) or was synchronous suggesting that the primary pathology originated in the lung. Petridis, et al. [3] described an unusual case with hemorrhagic brain lesions resembling cavernous hemangiomas. The patient had a fulminant course and died. It is not clear whether these co-existent brain lesions represent metastasis from a primary IMT or a multifocal inflammatory response of unknown etiology.

In our case, more than 10 brain lesions were observed. Maximum of three multifocal brain lesions have been reported in the literature. Another unusual aspect of our case was a presence of simultaneous bilateral lung lesions as well as the involvement of pulmonary vessels. Multiple lung lesions are seen in five percent of cases, with endobronchial masses constituting 10% of cases and only rare occurrence is observed in the pulmonary artery. On CT scans, pulmonary IMTs have a variable appearance, most commonly having heterogeneous attenuation and enhancement. Intralesional calcifications are more commonly seen in children. The pattern of calcification ranges from an amorphous, mixed, or fine fleck-like pattern to heavy mineralization. Cavitation and lymphadenopathy are rare. Atelectasis and pleural effusion may be seen [2]. Lesions in the brain may be solitary or multifocal and are usually well circumscribed solid and show enhancement. Calcification and necrosis are rare. Areas of reactive gliosis and neuronal loss may be present in the adjacent cortex [8].

Differential diagnosis of such multifocal, enhancing lesions is broad and primarily includes infections and neoplasms. Infective granulomas particularly tuberculomas are an important consideration, particularly in South Asia where the prevalence of tuberculosis is very high. Tumors like meningiomas and plasmacytomas may resemble central nervous system IMT but usually present in older age. Histology can reliably help in differentiating these from IMT. Other differentials include metastasis and central nervous system (CNS) lymphoma. Histoplasmosis X and Wegner's Granulomatosis may rarely cause brain lesions that mimic IMT [7-9].

## Conclusions

Inflammatory myofibroblastic tumor (IMT) in lung with simultaneous brain involvement is rare with variable clinical and radiological presentation. Patients with symptoms confined to one organ system should be carefully evaluated for synchronous tumors with high index of suspicion for common sites such as lungs, even in the presence of mild or no symptoms. Early diagnosis may render the cases operable which is the only established curative treatment.
